# Retinal capillary microvessel morphology changes are associated with vascular damage and dysfunction in cerebral small vessel disease

**DOI:** 10.1177/0271678X221135658

**Published:** 2022-10-27

**Authors:** Stewart J Wiseman, Jun-Fang Zhang, Calum Gray, Charlene Hamid, Maria del C Valdés Hernández, Lucia Ballerini, Michael J Thrippleton, Cameron Manning, Michael Stringer, Emilie Sleight, Susana Muñoz Maniega, Alasdair Morgan, Yajun Cheng, Carmen Arteaga, Dany Jaime Garcia, Una Clancy, Fergus N Doubal, Baljean Dhillon, Tom MacGillivray, Yun-Cheng Wu, Joanna M Wardlaw

**Affiliations:** 1 Centre for Clinical Brain Sciences, University of Edinburgh, Edinburgh, UK; 2 UK Dementia Research Institute, University of Edinburgh, Edinburgh, UK; 3 Edinburgh Imaging Facilities, Edinburgh Imaging, University of Edinburgh, UK; 4 Department of Neurology, Shanghai General Hospital, Shanghai Jiao Tong University School of Medicine, Shanghai, China; 5 Department of Neurology, West China Hospital, Sichuan University, Chengdu, China; 6 NHS Lothian Princess Alexandra Eye Pavilion, UK

**Keywords:** OCTA, vessel density, capillary branching, cerebrovascular reactivity, perivascular spaces

## Abstract

Cerebral small vessel disease (SVD) is a cause of stroke and dementia. Retinal capillary microvessels revealed by optical coherence tomography angiography (OCTA) are developmentally related to brain microvessels. We quantified retinal vessel density (VD) and branching complexity, investigating relationships with SVD lesions, white matter integrity on diffusion tensor imaging (DTI) and cerebrovascular reactivity (CVR) to CO_2_ in patients with minor stroke. We enrolled 123 patients (mean age 68.1 ± SD 9.9 years), 115 contributed retinal data. Right (R) and left (L) eyes are reported. After adjusting for age, eye disease, diabetes, blood pressure and image quality, lower VD remained associated with higher mean diffusivity (MD) (standardized β; R −0.16 [95%CI −0.32 to −0.01]) and lower CVR (L 0.17 [0.03 to 0.31] and R 0.19 [0.02 to 0.36]) in normal appearing white matter (NAWM). Sparser branching remained associated with sub-visible white matter damage shown by higher MD (R −0.24 [−0.08 to −0.40]), lower fractional anisotropy (FA) (L 0.17 [0.01 to 0.33]), and lower CVR (R 0.20 [0.02 to 0.38]) in NAWM. OCTA-derived metrics provide evidence of microvessel abnormalities that may underpin SVD lesions in the brain.

## Introduction

Cerebral small vessel disease (SVD) is a major cause of stroke and dementia yet its underlying mechanism remains unclear and treatments are limited. SVD pathophysiology is mainly thought to occur at the level of brain perforating microvessels and includes impaired vasodilation, subtle blood-brain barrier leakage and perivascular tissue damage,^1^ and vessel stiffening which could together impair glymphatic drainage.^2,3^ Common structural brain imaging features of SVD include white matter hyperintensities (WMH) and perivascular spaces (PVS).^4^ Magnetic resonance imaging (MRI) methods like blood oxygen level dependent (BOLD) cerebrovascular reactivity (CVR) imaging with hypercapnic stimulus^5^ and diffusion tensor imaging (DTI)^6^ can identify vascular dysfunction and white matter damage, respectively, in brain tissue.

Blood vessels in the retina share many features with similarly sized vessels in the brain, including their embryological origin and anatomic and physiological characteristics.^7^ By examining the condition of the retina’s microvascular system that is captured on non-invasive imaging, from the main arcades (∼40 μm in diameter) observed on fundus photography, to the capillaries (down to 8 μm) seen with newer technologies such as optical coherence tomography angiography (OCTA),^8^ we might draw useful conclusions about the brain’s microvascular health. OCTA might even be able to detect early microvascular changes before sequela are visible with brain imaging modalities.

Previous investigations with fundus photography have shown that patients with small vessel (lacunar) stroke have wider venules,^9^ smaller arterioles^10^ and decreased fractal dimension of the vessel branching pattern, suggesting a sub-optimal microvascular network compared with other ischemic stroke subtypes.^11^ Moreover, retinal microvascular abnormalities (such as focal arteriolar narrowing and arteriovenous nicking) observed on fundus imaging are associated with incident lacunar stroke during follow-up,^12^ and retinopathy (such as microaneurysms, haemorrhages and exudates) is associated with dementia^13^ and stroke.^14^

Markers of SVD in the brain have also been shown to associate with findings from fundus imaging. These include an increase in PVS volume associated with decreasing fractal dimension^15^ and increased retinal vessel branching coefficients with periventricular WMH.^16^

OCTA is a relatively recent advance in retinal imaging which visualizes retinal capillaries, and is described as having ‘near histology level resolution’^8^ (see Kashani et al.^8^ for its application in eye disease). In neurological disease, a recent systematic review^17^ found changes on OCTA in the foveal avascular zone, perfusion density and vessel density (VD) in patients with Alzheimer’s disease (AD) and pre-symptomatic AD but sparse data precluded conclusion about OCTA findings in SVD and whether decreased VD was associated with worse WMH. There was no information on other SVD features such as PVS and few studies accounted for covariates. Therefore, whether and how OCTA imaging features relate to brain imaging features in sporadic SVD remains unclear.

Here, we investigated associations between retinal microvascular parameters measured using OCTA and SVD markers from brain MRI including visible SVD lesions, blood flow, cerebrovascular reactivity, and DTI measurements of white matter microstructural integrity in patients with SVD who presented with a minor stroke.

## Materials and methods

### Participants

We recruited adult patients with mild (i.e., expected to be non-disabling) ischemic stroke (Modified Rankin Scale ≤2) from NHS Lothian clinical stroke services into the Mild Stroke Study 3 (MSS-3; ISRCTN registry 12113543). We excluded patients with contraindications to MRI, incapacity to consent or severe cardiac failure or respiratory disease. Assessments included clinical, lifestyle and cognitive assessments, blood tests and brain and retinal imaging at baseline (up to 3 months after stroke), three months, six months and one-year. The rationale for the study along with the protocol and design of this single centre, observational, longitudinal cohort study and details of data collection have been published.^18^

Here we focus on the baseline visit in which retinal imaging and brain MRI were conducted on the same day. The South East Scotland Research Ethics Committee (18/SS/0044) approved the study and all participants gave written informed consent. All procedures were conducted according to the Declaration of Helsinki of 1975 (and as revised in 1983).

### Vascular risk factors

Medical histories including cardiovascular risk factors such as smoking status, previous stroke, hypertension, hypercholesterolemia, and diabetes mellitus and currently prescribed medications were recorded.^18^ All patients received guideline secondary stroke prevention (antiplatelet, antihypertensive and lipid lowering agents). Resting blood pressure readings were recorded.

### MRI brain image acquisition

Full details of the brain imaging scanning protocol for MSS-3 have been published.^18^ Briefly, participants underwent brain MRI at 3T (MAGNETOM Prisma, Siemens Healthcare GmbH, Erlangen, Germany) with the following sequences: 3D T1-weighted (1 mm^3^ isotropic; TR 2500; TE 4.37), T2-weighted (0.9 mm^3^ isotropic; TR 3200; TE 408), fluid attenuated inversion recovery-weighted (FLAIR) (1 mm^3^ isotropic; TR 5000; TE 388; TI 1100), susceptibility-weighted (SWI) (0.6 × 0.6 × 3 mm; TR 28; TE 20), inversion recovery spin gradient sequence (IR_SPGR) and multi-shell diffusion MRI. At the same visit, we also measured BOLD response to 6% CO_2_ as a standardized measure of cerebrovascular reactivity (CVR) (see below), and total blood through the internal carotid and vertebral arteries to calculate mean arterial blood flow across the cardiac cycle and total cerebral blood flow (CBF).^18^

### Analysis of MRI data

- Visual rating of SVD

All MRI visual assessments of SVD were conducted with reference to STRIVE guidelines.^4^ We used the Fazekas^19^ scale to visually rate WMH and validated tools to grade PVS in the centrum semiovale (CSO PVS) and basal ganglia (BG PVS).^20,21^ Further details are in Supplementary Material.

- Intracranial volume, brain tissue volumes and quantitative image analysis of WMH and PVS

All image sequences were co-registered to the T2-weighted sequence and processed with FLIRT,^22^ FSL,^23^ BET2^24^ and in-house software as described in full in Supplementary Material.

- Arterial blood flow to the brain

We manually segmented the internal carotid and vertebral arteries on the phase contrast maximum magnitude image using FSLEyes (https://fsl.fmrib.ox.ac.uk/fsl/fslwiki/FSLeyes) and corrected for background phase error.^25,26^ Here, we compare mean arterial blood flow across the cardiac cycle (ml/s) in relation to OCTA metrics.

- Cerebrovascular reactivity of brain vessels

We assessed CVR using a block design gas challenge alternating between medical air (2 minutes) and air with 6% carbon dioxide (CO_2_) (3 minutes) using an established protocol.^5,27–29^ We calculated two metrics using linear regression of the mean BOLD signal in NAWM against the end tidal CO_2_ with a variable delay and controlling for drift using in-house MATLAB code as previously described.^5^ CVR magnitude is the amplitude of the response (higher indicates a stronger vasodilatory response) given in % BOLD signal change per mmHg change in end tidal CO_2_ (%/mmHg). CVR measurements analyzed here are derived from NAWM masks.

- Diffusion imaging to assess white matter integrity

All WMH and NAWM diffusion measurements were computed in diffusion space as described in full in Supplementary Material.

### Retinal imaging

The retinal examination used spectral domain optical coherence tomography (OCT) with eye tracking technology (SPECTRALIS OCT2, Heidelberg Engineering, Germany) with both left and right eyes imaged.^18^ We recorded self-declared eye disease (diabetic retinopathy, glaucoma, and age-related macular degeneration) which we checked against our ophthalmological graded assessments of the images. Queries were resolved by an ophthalmologist (B.D.).

### OCTA image acquisition

The macula was localized by a scanning laser ophthalmoscope infrared fundus image. Next, a 10° × 10° structural OCT image was captured together with the corresponding OCTA flow data, detected by variation in acquired signal and representing the movement of red blood cells through vessels.^8^ The scan pattern in MSS-3 consists of 512 B-Scans separated at 6 μm intervals. The combined structural OCT and functional OCTA covers 3 mm × 3 mm of the retina, centered at the fovea.

### OCTA image processing

**Figure 1.**
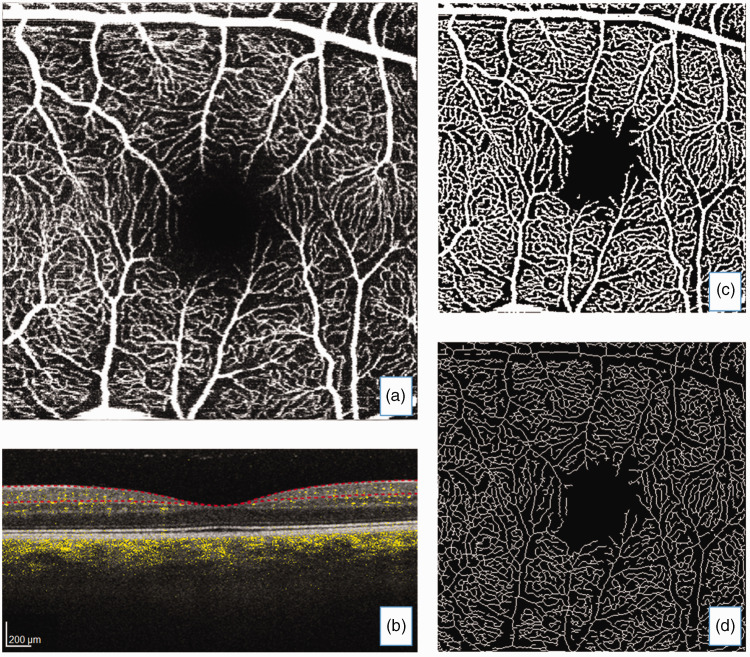
(a) *Enface* optical coherence tomography angiography (OCTA) transverse image of the superficial vascular complex with (b) corresponding structural and OCTA flow image where yellow colouration represents blood flow in vessels and segmentation (red dotted lines) bounds the internal limiting membrane to inner plexiform layer. Processed images: (c) adaptive threshold with Hessian filter and (d) skeletonized image.

All image analysis was performed masked to patient characteristics and all other data (i.e., retinal imaging masked to MRI and clinical data, and vice versa). *Enface* transverse images of the superficial vascular complex (SVC) were extracted – i.e., vessels bounded by the internal limiting membrane to inner plexiform layer, being the vessels that supply blood to the retinal nerve fiber layer and ganglion cells (Figure 1). These images were processed using bespoke software written in MATLAB (MathWorks, Natik, MA, USA; version 2016a).

### Vessel density and vessel branching complexity

VD was calculated from a binary map of the vessels and indicates the percentage of vessel area (white pixels to black background) occupying the image.^30^ To create the binary map a pre-processing step was performed using Hessian-based Frangi filters to enhance vessel structures. Next, the filtered image was thresholded using Otsu (‘global’) and local (‘adaptive’) thresholding methods. The outputs from both methods were combined to generate the final binary map that contained vessels where both threshold images agreed. Larger values of VD reflect greater density.

Fractal analysis quantifies the complexity of the vessel branching pattern visible in a retinal image in terms of fractal dimension, being a single value that summarizes how complex or sparse the vascular pattern is.^30,31^ We used a multifractal/multispectral approach that models the vascular pattern in the binary map as if composed of many fractals with different fractal dimensions depending on the scale,^32^ yielding a spectrum of fractal dimensions.^33^ Values are unitless, with larger numbers reflecting greater complexity.

### Quality of OCTA images

Image quality can be affected by patient movement and media opacities such as cataracts which adversely impact the measurement process. The manufacturer’s OCTA quality index, Q, being the average signal strength measured in decibels for all structural B-scans in the image volume, was recorded for each eye. B-scans of Q < 20 are rejected during acquisition, thus the averaged Q has a lower bound of 20. Healthy eyes with no media opacities can achieve Q values of around 40 or higher, thus Q tends to range from 20 to 40. Heidelberg Engineering suggests good OCTA *enface* images are obtained with Q > = 28.

Q does not always correlate well with image quality as perceived by a human rater. Important factors not captured in Q include focus and residual eye motion. Thus, two trained raters (S.J.W. and J.F.Z.) visually graded the interpretability of all OCTA images independently on an ordinal scale with 5 categories (1 = “Inadequate”, 2 = “Poor”, 3 = “Average”, 4 = “Good” and 5 = “Excellent”) (examples given in Supplementary Figure 1), and disagreements were resolved by discussion. Agreement between raters was determined by intraclass correlation coefficient (ICC)^34^ with estimates calculated using the ‘irr’ package.^35^ The final consensus in visual assessment was then compared to Q using Spearman’s correlation.

### OCTA image quality: inter-rater visual agreement and correlation with signal strength

Rater agreement in OCTA image quality visual assessment was ICC 0.71 [95%CI 0.53 to 0.82] and ICC 0.69 [0.41 to 0.83] for right and left eyes respectively. Spearman’s correlation between the visual assessment consensus and the instrument manufacturer’s signal strength Q index was *r* = 0.62 and *r* = 0.56 for right and left eyes, respectively. We account for image quality in the primary analyses by adding it as a covariate in the adjusted models.

### Statistical analysis

Data were checked for normality by visual inspection of the distributions and by Kolmogorov-Smirnov test. We log transformed WMH volumes. Missing data were recorded (Table 1). All analyses were per patient, and although we analyze both right and left eyes, we do so as separate tasks, and show results from both eyes for transparency. Eye asymmetry could be important.^36^

**Table 1.** Mild Stroke Study 3 demographics, OCTA data, co-morbid eye disease, vascular risks, and comprehensive brain imaging data at baseline.

Mean age (years)	68.1 (SD 9.9)	
Male/Female	80/43	
OCTA	*Right eye (N* = *104)*	*Left eye (N* = *105)*
Vessel density (%)	32.1 (SD 5.62)	32.1 (SD 5.24)
Fractal complexity (unitless)	1.81 (SD 0.03)	1.82 (0.03)
OCTA image quality		
Signal strength ‘Q’ (decibels)	37.3 (SD 2.92)	37.7 (SD 2.90)
1 Inadequate (N)	12	9
2 Poor (N)	24	31
3 Average (N)	52	47
4 Good (N)	6	10
5 Excellent (N)	10	8
Co-morbid eye disease	*N* = *117*	
Diabetic retinopathy	6/117 (5.13%)	
Glaucoma	3/117 (2.56%)	
AMD	5/117 (4.27%)	
Any of the above	14/117 (11.96%)	
Vascular risk factors	*N* = *123*	
Diabetes	27/123 (21.9%)	
Hypertension	87/123 (70.7%)	
Hypercholesterolemia	92/123 (74.7%)	
Smoker: current	14/123 (11.4%)	
Smoker: never *v.* ever	56/123 (45.5%) *v.* 67/123 (54.4%)	
Mean SBP (mmHg)	149.0 ± 19.67	
White matter lesions	*N* = *122*	*% of ICV*
ICV volume (ml)	1,612.0 (SD 160.4)	
WMH volume, median (ml)	8.17 (3.47–18.60)	0.50
Fazekas periventricular (0:3)	1.5 (1–2)	
Fazekas deep (0:3)	1 (1–2)	
Fazekas total (0:6)	3 (2–3)	
PVS: computational	*N* = *115*	*% of CSO or BG* ^a^
PVS volume in CSO (ml)	11.5 (6.9–17.5)	3.6 (2.2–5.8)
PVS volume in BG (ml)	2.9 (2.1–3.8)	4.9 (3.7–6.0)
PVS count in CSO	890 (302)	
PVS count in BG	170 (41)	
PVS: visual ratings	*N* = *122*	
CSO PVS	3 (2–3)	
BG PVS	2 (1–2)	
Blood flow to the brain	*N* = *100*	
Arterial mean (ml/s)	9.44 (SD 1.94)	
Cerebrovascular reactivity	*N* = *114*	
CVR magnitude (%/mmHg)	0.043 (SD 0.020)	
CVR delay (s)	30.89 (SD 21.0)	
White matter integrity	*N* = *117*	
FA in NAWM (unitless)	0.42 (SD 0.02)	
FA in WMH (unitless)	0.27 (SD 0.04)	
MD in NAWM (10^–3^ mm^2^/s)	0.76 (SD 0.02)	
MD in WMH (10^–3^ mm^2^/s)	1.09 (SD 0.07)	

AMD: age-related macular degeneration. BG: basal ganglia. CSO: centrum semiovale. CVR: cerebrovascular reactivity. FA: fractional anisotropy. ICV: inter cranial volume. MD: mean diffusivity. NAWM: normal appearing white matter. PVS: perivascular spaces. SBP: systolic blood pressure. WMH: white matter hyperintensity.

^a^PVS volume as a percentage of the region of interest, either CSO or BG. ICV volume and PVS counts as mean (SD), other volumes have non-normal distributions and so presented as medians (interquartile range, Q1–Q3).

WMH volumes were corrected for ICV volume, and PVS volumes corrected for the relevant region of interest volume, either centrum semiovale or basal ganglia. PVS counts are given as counts without correction for a region of interest.

We used linear regression to investigate univariate associations between VD and branching pattern fractal complexity as outcomes of interest with various predictor variables: age and sex, vascular risks (smoking, blood pressure measures, hypertension, diabetes, high cholesterol) and various brain MRI measurements. Next, multiple general linear models estimated the relationship between OCTA variables and brain imaging variables, controlling for key co-variates: comorbid eye disease, age, diabetes, blood pressure and OCTA image quality. Model estimates for the continuous predictors are presented as standardized betas (β). All statistical analyses were conducted in R Studio using R version 3.6.1.

## Results

We analyzed data from the baseline visit obtained on average 53 days (range 6–95 days) after index stroke symptoms in 123 consecutive participants recruited to MSS-3 between August 2018 and August 2020. Mean age across the cohort was 68.1 ± SD 9.9 years (range 40–86 years).

There were 80/123 (65%) men, 14/123 (11.4%) smokers, 27/123 (21.9%) had diabetes and 87/123 (70.7%) were hypertensive. Median WMH volume was 8.17 ml (interquartile range: 3.47–18.60). Patient-level missing data were due to technical and organizational issues with retinal image acquisition (N = 8) leaving N = 115 participants with retinal examinations (mean age of these 115 was 68.2 ± SD 10.0 years). Eleven right and 10 left eye OCTAs were not performed or were unsuccessful due to severe eye disease, leaving 104 right and 105 left eyes for the current analyses.

Patient demographics and baseline retinal and brain imaging measurements are given in Table 1. One participant did not have a baseline brain scan due to MRI claustrophobia but is retained in the analysis to contribute to the summarized vascular risks and eye data in Table 1. VD and branching complexity (i.e., multispectral fractal dimension) were correlated: right eye *r* = 0.93 and left eye *r* = 0.90.

### OCTA retinal vessel density

We analyze and report right (R) and left (L) eyes separately. On univariate analyses, lower VD related to older age, hypertension, diabetes, and comorbid eye disease, as well as more WMH, more CSO and BG PVS, and MD both in NAWM and in WMH (Supplementary Figure S2). As expected, higher VD related to better image quality, higher CVR magnitude and higher FA in NAWM (Supplementary Figure S2).

**Figure 2.**
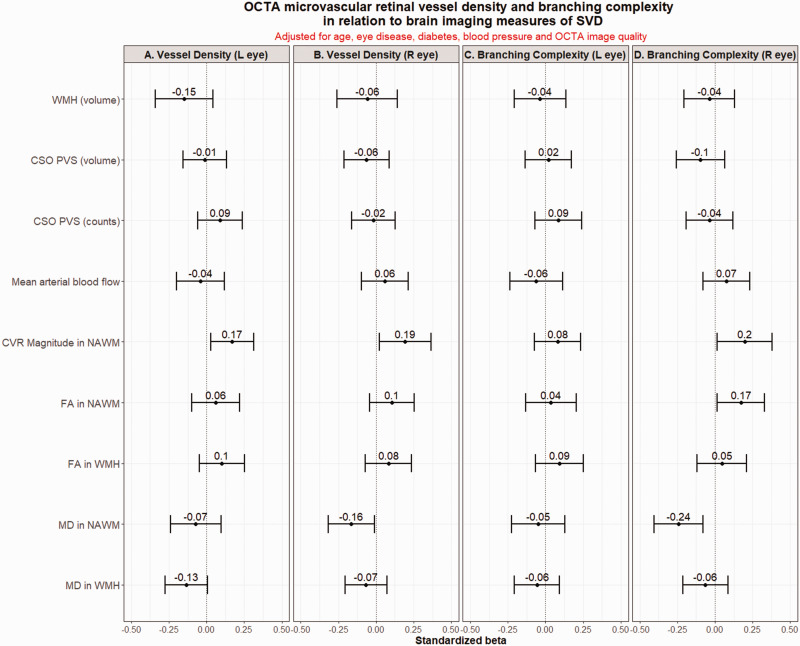
Fully adjusted models. OCTA vessel density (panels a and b) and vessel branching complexity (panels c and d) in relation to brain imaging variables characterizing SVD, after adjustment for age, eye disease, diabetes, systolic blood pressure and OCTA image quality. CSO PVS: centrum semiovale perivascular spaces; CVR: cerebrovascular reactivity; FA: fractional anisotropy; MD: mean diffusivity; OCTA: optical coherence tomography angiography; SVD: small vessel disease; WMH: white matter hyperintensities.

After adjusting for age, eye disease, diabetes, blood pressure and image quality, lower VD remained associated with higher MD (standardized β; R −0.16 [95%CI −0.32 to −0.01]) and lower CVR (L 0.17 [0.03 to 0.31] and R 0.19 [0.02 to 0.36]) in NAWM (Figure 2). We did not find any association between VD and arterial blood flow.

### OCTA retinal vessel branching complexity (fractal dimension)

On univariate analyses, less complex branching related to older age, diabetes, comorbid eye disease, greater WMH burden, more CSO PVS and BG PVS and higher MD in NAWM (Supplementary Figure S3). More complex branching related to better image quality and higher FA in NAWM (Supplementary Figure S3). After adjusting for covariates, sparser branching associated with higher MD (R −0.24 [−0.08 to −0.40]) and lower FA (L 0.17 [0.01 to 0.33]), and lower CVR (R 0.20 [0.02 to 0.38]) in NAWM (Figure 2). We did not find any association between branching complexity and arterial blood flow.

## Discussion

This study demonstrates that lower retinal VD on OCTA is associated with higher MD and lower CVR magnitude in NAWM, consistent with a reduction in small vessel density in the brain underpinning the observed impairments in vascular function and sub-visible white matter structure seen with increasing SVD lesion burden. We also observed lower branching complexity (fractal dimension) with lower CVR magnitude, and with sub-visible white matter damage suggested by higher MD and lower FA in NAWM.

CVR estimates the adequacy of the change in blood flow in response to a vasoactive stimulus, and is impaired in SVD particularly in affected tissues.^29^ CVR magnitude in white matter is lower in patients with more severe SVD features,^28^ and CVR delay is longer in SVD patients than healthy controls.^5,37,38^ Here, we found that lower retinal VD on OCTA was associated with lower CVR magnitude in NAWM after correcting for a range of key covariates, also validating the results of a much smaller (N = 11) study of older subjects.^39^ Lower branching complexity related to lower CVR and higher MD in NAWM.

Increased MD occurs in WMH and also in NAWM as the severity of SVD lesions increase and indicates sub-visible alterations in white matter integrity.^40^ No prior studies investigated the relationship between OCTA parameters and DTI measures of white matter structural integrity in SVD. Here, we found that lower retinal VD on OCTA was associated with increased MD in NAWM, consistent with vessel density decreasing in NAWM as the SVD burden worsens. Here, reduced branching complexity also related to more sub-visible white matter damage (higher MD and lower FA in NAWM) in keeping with increased interstitial fluid content and mobility, demyelination, and axonal loss. It will be important to assess whether early retinal vascular changes can predict WMH progression and white matter damage in the long term.

PVS in the brain are a marker of vascular dysfunction, amongst several associations, and occur early in development of SVD.^41^ We have previously demonstrated a link between increasing PVS volumes and decreasing fractal dimension of larger retinal arterioles on fundus photography,^15^ as did a previous study of PVS in the CSO.^42^ Here, there were strong univariate associations between both lower VD and less complex branching and more PVS (computational volumes and counts but not visual ratings), but none survived adjustment for covariates. The typical vessel arborization pattern of the retina is thought to be in accordance with Murray’s Law of Minimal Work, which strives for less energy dissipation and maximum efficiency of energy use when transporting blood.^43^ Reduction from the optimized vessel branching to sparser patterns may link to SVD lesions through impaired small vessel function (as reflected in impaired CVR) and less efficient energy management.

WMH are a common feature of cerebral SVD on brain MRI. Previous retinal OCTA studies used Fazekas score to represent WMH severity, with inconsistent findings.^17,39,44,45^ In a wide range of study populations including AD, mild cognitive impairment (MCI), SVD or cognitively normal adults, some studies^17,39,44^ observed that decreased VD on OCTA was associated with increased Fazekas score, while others^17,45^ showed no significant association which might partly be due to small sample size.^17^ Here, worse WMH (computationally and by visual score) strongly related to reduced VD and reduced branching on univariate analyses (Supplementary Figure S2) but effects were lost in fully adjusted analyses.

In our study, we did not find an association between OCTA measures and resting arterial blood flow, possibly suggesting retinal/central perfusion stability. In a small study (35 eyes from 18 patients) of pre- and post-operative major non-neurological surgery requiring planned intensive therapy unit (ITU) admission,^46^ retinal blood flow was stable and the authors conclude that the stability of retinal blood flow measures suggests the potential for OCTA to provide clinically useful measures in ITU patients. OCTA could be used as a potential biomarker of central perfusion in SVD but more data is needed. In a recent updated meta-analysis,^47^ WMH were found to associate with reduced cerebral blood flow cross-sectionally but when studies of patients with dementia or that failed to account for vascular risk factors were excluded, the relationship disappeared. Longitudinal associations between blood flow and SVD burden are mixed and remain unclear.^47^

The strengths of this study are the relatively large sample of patients with a confirmed stroke, analyses of both eyes per participant, a comprehensive brain imaging protocol that generated key brain imaging metrics describing SVD, and adjustment for vascular risk factors and OCTA image quality. We also acknowledge weaknesses. The cross-sectional design precludes inferences on the temporal link between impaired retinal microvasculature and SVD progression but MSS-3 is gathering the data to address retinal and brain changes in SVD. We did not assess retinal vessels from the deep vascular layers. There are no data in our study representing a non-stroke control group, rather, we are interested in within-group associations between the eye and brain to help deepen our understanding of small vessel dysfunction.

In conclusion, our study showed that impaired retinal capillary-level changes identified using OCTA were associated with structural SVD markers and impaired cerebral vasoreactivity. OCTA measures hold promise as non-invasive biomarkers of cerebral SVD, being relatively easy to tolerate and economical. Longitudinal studies are warranted to assess the predictive power of OCTA measurements for SVD progression and monitor treatment responses.

## Supplemental Material

sj-pdf-1-jcb-10.1177_0271678X221135658 - Supplemental material for Retinal capillary microvessel morphology changes are associated with vascular damage and dysfunction in cerebral small vessel disease Click here for additional data file. Supplemental material, sj-pdf-1-jcb-10.1177_0271678X221135658 for Retinal capillary microvessel morphology changes are associated with vascular damage and dysfunction in cerebral small vessel disease by Stewart J Wiseman, Jun-Fang Zhang, Calum Gray, Charlene Hamid, Maria del C Valdés Hernández, Lucia Ballerini, Michael J Thrippleton, Cameron Manning, Michael Stringer, Emilie Sleight, Susana Muñoz Maniega, Alasdair Morgan, Yajun Cheng, Carmen Arteaga, Dany Jaime Garcia, Una Clancy, Fergus N Doubal, Baljean Dhillon, Tom MacGillivray, Yun-Cheng Wu and Joanna M Wardlaw in Journal of Cerebral Blood Flow & Metabolism

## Open access publishing statement

This research was funded in whole, or in part, by the Wellcome Trust [Grant number 104916/Z/14/Z]. For the purpose of open access, the author has applied a CC BY public copyright license to any Author Accepted Manuscript version arising from this submission.
