# Supplementary material for: Retinal capillary microvessel morphology changes are associated with vascular damage and dysfunction in cerebral small vessel disease
Source: J Cereb Blood Flow Metab. 2022 Oct 27;43(2):231–40. doi: 10.1177/0271678X221135658 (PMC9903216; doi:10.1177/0271678X221135658)
Supplement: sj-pdf-1-jcb-10.1177_0271678X221135658 - Supplemental material for Retinal capillary microvessel morphology changes are associated with vascular damage and dysfunction in cerebral small vessel disease [file sj-pdf-1-jcb-10.1177_0271678X221135658.pdf]

Supplementary Materials

Retinal capillary microvessel morphology changes are associated with vascular damage and dysfunction in cerebral small vessel disease

Stewart J. Wiseman[a,b,c]*, Jun-Fang Zhang[d]*, Calum Gray[c], Charlene Hamid[a,c], Maria del C. Valdés Hernández[a,b], Lucia Ballerini[a,b], Michael J. Thrippleton[a,b,c], Cameron Manning[a], Michael Stringer[a,b,c], Emilie Sleight[a,b], Susana Muñoz Maniega[a], Alasdair Morgan[a], Yajun Cheng[a], Carmen Arteaga[a], Dany Jaime Garcia[a], Una Clancy[a], Fergus N. Doubal[a], Baljean Dhillon[a,f], Tom MacGillivray[a,c], Yun-Cheng Wu[d], Joanna M. Wardlaw[a,b,c]

[a] Centre for Clinical Brain Sciences, University of Edinburgh, UK
[b] UK Dementia Research Institute, University of Edinburgh, UK
[c] Edinburgh Imaging Facilities, Edinburgh Imaging, University of Edinburgh, UK
[d] Department of Neurology, Shanghai General Hospital, Shanghai Jiao Tong University School of Medicine, Shanghai, China
[e] Department of Neurology, West China Hospital, Sichuan University, Chengdu, China
[f] NHS Lothian Princess Alexandra Eye Pavilion, UK

* These authors contributed equally to this work

Supplementary Methods

Analysis of MRI data

- **Visual rating of SVD**

All MRI visual assessments of SVD were conducted with reference to STRIVE guidelines[1]. Deep and periventricular WMHs were coded 0 to 3 using the Fazekas[2] scale and summed to give a total WMH score (0–6) per subject. Visible (i.e., enlarged) PVS are round (<3 mm) or linear depending on the scan plane in relation to the orientation of the vessel[3] and their intensity is that of cerebrospinal fluid on T2-weighted scans. They were assessed in the centrum semiovale (CSO PVS) and basal ganglia (BG PVS) and scored as 0 (none), 1 (1–10 PVS), 2 (11–20), 3 (21–40) and 4 (>40) using a validated scale[3,4]. We note that not all enlarged PVS are necessarily visible in MRI, and by implication dysfunctional, based solely on their conspicuity at a macro-scale. However, MRI-visible PVS appear mostly clinically relevant[5], as studies have reported their number and volume overall to increase with age, vascular risk factors (particularly hypertension) and other features of small vessel disease.

- **Intracranial volume, brain tissue volumes and quantitative image analysis of WMH and PVS**

All image sequences were co-registered to the T2-weighted (T2W) image using FLIRT[6] from the FMRIB Software Library (FSL[7]). The intracranial volume (ICV) was generated automatically from the co-registered IR_SPGR image using BET2[8] from the same software library, and checked and manually edited if necessary. Normal appearing white matter (NAWM) was generated automatically after combining the outputs from FSL-FAST[7] and Freesurfer (https://surfer.nmr.mgh.harvard.edu/), both run using the manually-corrected ICV**.** A raw probabilistic mask of normal appearing white matter (NAWM) was generated from FSL-

FAST (ver 6.0) run with the default parameters using the T1-weighted (T1W) image, after being transformed to the T2W native space using FSL-FLIRT (also run with the default parameters), corrected for bias-field inhomogeneities, and brain-extracted using the manually-edited ICV. Freesurfer (ver 6.0) full processing stream (i.e., “recon-all”) was run using as input the T1W and the T2W images, both in the T2W space. The raw NAWM mask from FSL-FAST was further cleaned with the output from Freesurfer by multiplying it by the binarized NAWM region obtained from Freesurfer. Any inaccuracies were also corrected using the meningeal and pial binary masks generated from Gaussian clustering[9] a multispectral space containing the brain-extracted T1-, T2- and FLAIR-weighted sequences, followed by the expectation-maximization algorithm.

A quantitative measure of WMH volume (in ml) was derived from hierarchically thresholding the T2-weighted-registered FLAIR image and removing false positives using the Freesurfer output. Hyperintense voxels on FLAIR were identified by thresholding intensity values 1.69 times the standard deviation above the mean normalised intensity of the brain tissue in this sequence[10]. Artefacts and hyperintense areas in the cortex were removed automatically using a lesion distribution probabilistic atlas derived from more than 600 MRI brains previously processed from ageing and SVD studies. Further refinement was achieved by applying Gaussian smoothing, and removing voxels with intensity values below 0.1 and z-scores below 0.95. Artefactual hyperintensities in and around the choroid plexus, third ventricle and cisterna magna were removed using the indexed segmentation output from Freesurfer (i.e., file labelled “aseg.auto”). All acute and old stroke lesions were manually drawn on the same FLAIR sequence by one experienced rater (S.J.W.), guided by the other MRI sequences, particularly diffusion restriction, and discussed with a neuroradiologist (J.M.W.). The total hyperintensities’ binary masks were carefully inspected and manually corrected for artefact-

induced false positives that could have been missed by the automatic pipeline and stroke lesions to generate the WMH binary masks. Stroke masks are deducted from the hyperintensities' masks such that final WMH mask volumes are not inflated.

PVS volumes (in ml) and PVS counts were calculated using a computational method described in full previously[11,12]. Performance of the computational method is better when the T2-weighted input images are free from gross movement artefact and so two experienced raters (L.B. and M.V.H.) independently assessed each image then agreed a final set prior to PVS quantification (seven brain images were classified as too noisy and so excluded from volumetric PVS quantification). The PVS segmentation method uses a vesselness filter optimised to generate the likelihood of a group of T2-hyperintense voxels of being a tubular structure or not[11]. This output is normalised and thresholded to generate the PVS binary mask. Therefore, assuring that the WMH of different shape, intensity level and size, are not mistaken by PVS and erroneously included in the segmentation. Further visual check of all masks to avoid confounds with small WMH and lacunes was done independently by three observers (M.V.H., Y.C., and L.B.). Lacunes mistakenly considered as PVS were manually excluded. PVS inside WMH were detected following the same procedure but using a fused image obtained from subtracting the FLAIR from the T2-weighted after both being bias field corrected[13].

To adjust for varying region of interest sizes across participants, WMH volume is adjusted for head size by dividing the WMH volume by the ICV volume, and PVS volumes are expressed as a percentage of the region of interest volume, i.e., either CSO or BG masks. CSO and BG masks were computed by extracting the NAWM and corpus striatum structures, respectively, from the Freesurfer automatic segmentation files "filled" and "aseg.auto", respectively, after these being mapped back to the native T2W space. These ROIs were cleaned for inaccuracies

using the output from a multispectral Gaussian clustering operating on a 4D array formed by the concatenation of T1W, T2W and FLAIR. Corpus striatum structures were merged to conform the BG ROI using binary morphological operations of dilation, opening and closing.

- **Diffusion imaging to assess white matter integrity**

The magnitude and directionality of water molecules diffusion can be quantified using mean diffusivity (MD) and fractional anisotropy (FA) respectively, with low MD and high FA characteristic of structurally intact white matter. Diffusion data was read and converted from DICOM to NIfTI-1 format and processed using TractoR v 3.3.5 'dpreproc' pipeline (http://www.tractor-mri.org.uk)[14]. Briefly, volumes were masked using FSL's brain extraction tool[15] and corrected for susceptibility and eddy current induced distortions using 'topup' and 'eddy' from FSL version 6.0.1[16,17]. In order to fit a diffusion tensor model, we analyzed only the diffusion-weighted volumes equivalent to a single-shell acquisition, with 11 volumes at b=0 and 64 volumes at b=1000 s/mm^2. Diffusion tensors were fitted at each voxel using FSL's 'dtifit', with weighted least squares estimation, and water diffusion maps were estimated for MD and FA[18].

All WMH and NAWM diffusion measurements were computed in diffusion space. For each participant, the 3D T2-weighted volume was registered to the averaged diffusion S0 volume with the TractoR functions 'reg-linear' followed by 'reg-nonlinear'. The transformation was then applied to WMH and NAWM masks and the median diffusion parameters obtained per participant.

QC of the PVS segmentation

PVS segmentation has three stages: 1) preprocessing, 2) filtering, 3) thresholding. The preprocessing stage involves bias field correction, intensity normalization and selection of the

Regions of Interest (ROI). ROI were all visually checked. Confounding voxels in the boundary between the subarachnoid space and the brain tissue were consistently missed out from the centrum semiovale (CSO) ROI. Strings of chronic PVS in the external capsule that could be missed by the automatic ROI segmentation, were manually included afterwards. Influence and robustness of the vesselness filters in the results of the segmentation have been evaluated already using a digital phantom[19]. We evaluated the influence of threshold selection in our segmentations, which is selected by the image analyst after visual inspection. We compared a first set of thresholds (i.e., one for the BG ROI and another for the CSO ROI) that was more conservative, against a second set of thresholds (labelled as “NEW” in Supplementary Figure S4) thought to be more selective in the CSO while being more inclusive in the BG. Both sets of thresholds were agreed by the image analysts, and segmentation results were compared. Supplementary Figure S4 shows the difference between the results obtained using each of them. The raw Pearson’s correlation between both sets of segmentations was 0.978 for the CSO PVS counts, 0.906 for the CSO PVS volume, 0.912 for the BG PVS counts, and 0.757 for the BG PVS volume (See Supplementary Figure S5). Associations were unchanged regardless of the threshold set used. Comparison between the PVS counts and volumes assessed only in the normal-appearing tissue vs. those assessed in the entire ROIs can be seen in Supplementary Figure S6. Examples of segmented PVS are given for low burden (Supplementary Figure S7) and high burden (Supplementary Figure 8).

Supplementary Figures

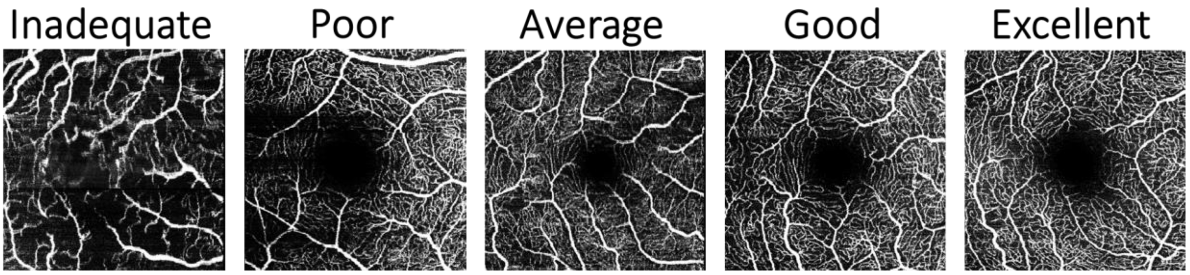

Supplementary Figure S1. Enface optical coherence tomography angiography (OCTA) transverse images with various gradations of perceived quality. We account for image quality by including the manufacturer's Q value as a covariate in our adjusted models.

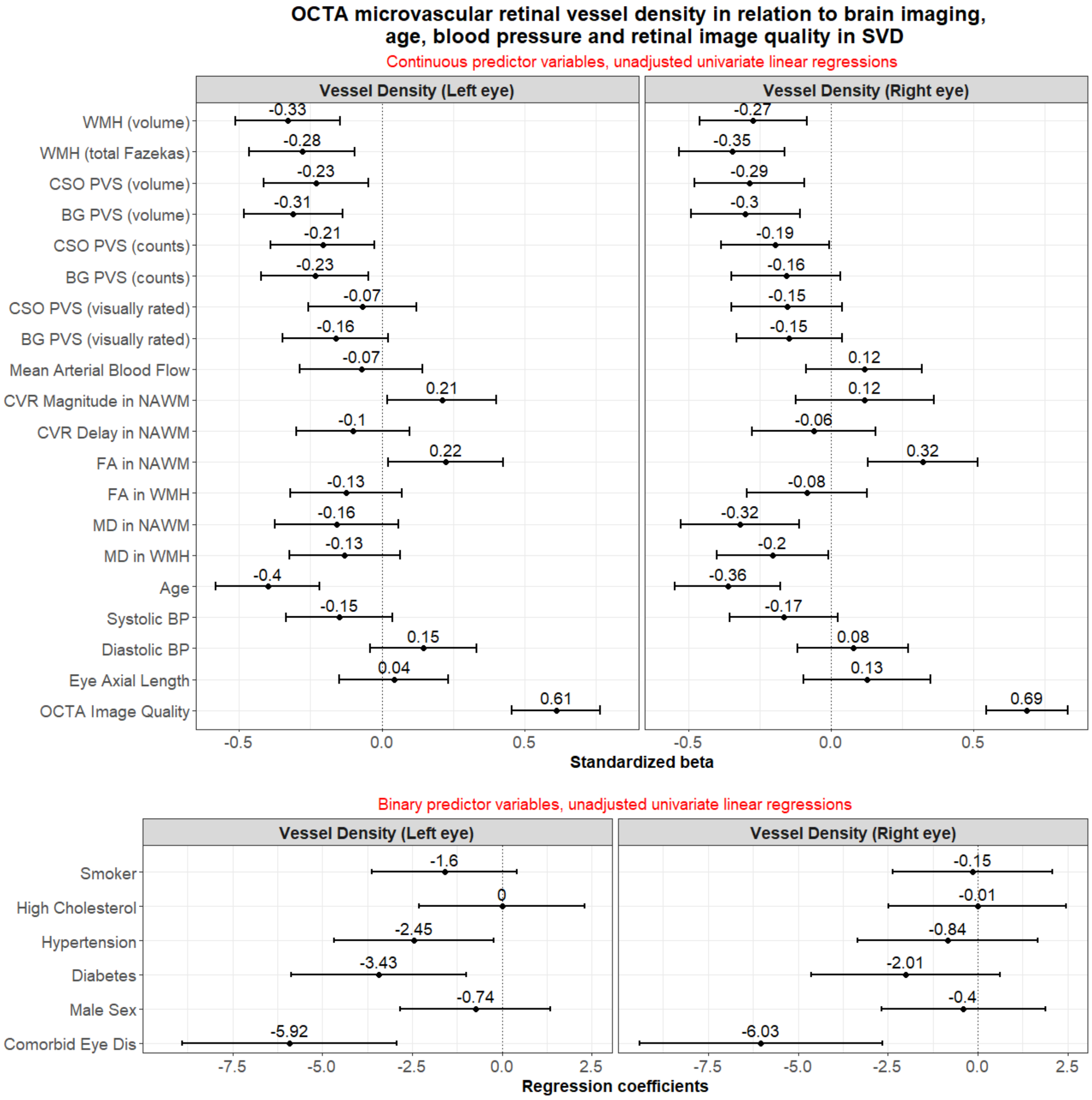

Supplementary Figure S2. Unadjusted univariate models. OCTA vessel density in relationship to age, sex, vascular risks and brain imaging measures of SVD showing standardized betas for continuous predictors and regression coefficients for binary predictors. *BG PVS = basal ganglia perivascular spaces; CSO PVS = centrum semiovale perivascular spaces; CVR = cerebrovascular reactivity; FA = fractional anisotropy; MD = mean diffusivity; OCTA = optical coherence tomography angiography; SVD = small vessel disease; WMH = white matter hyperintensities.*

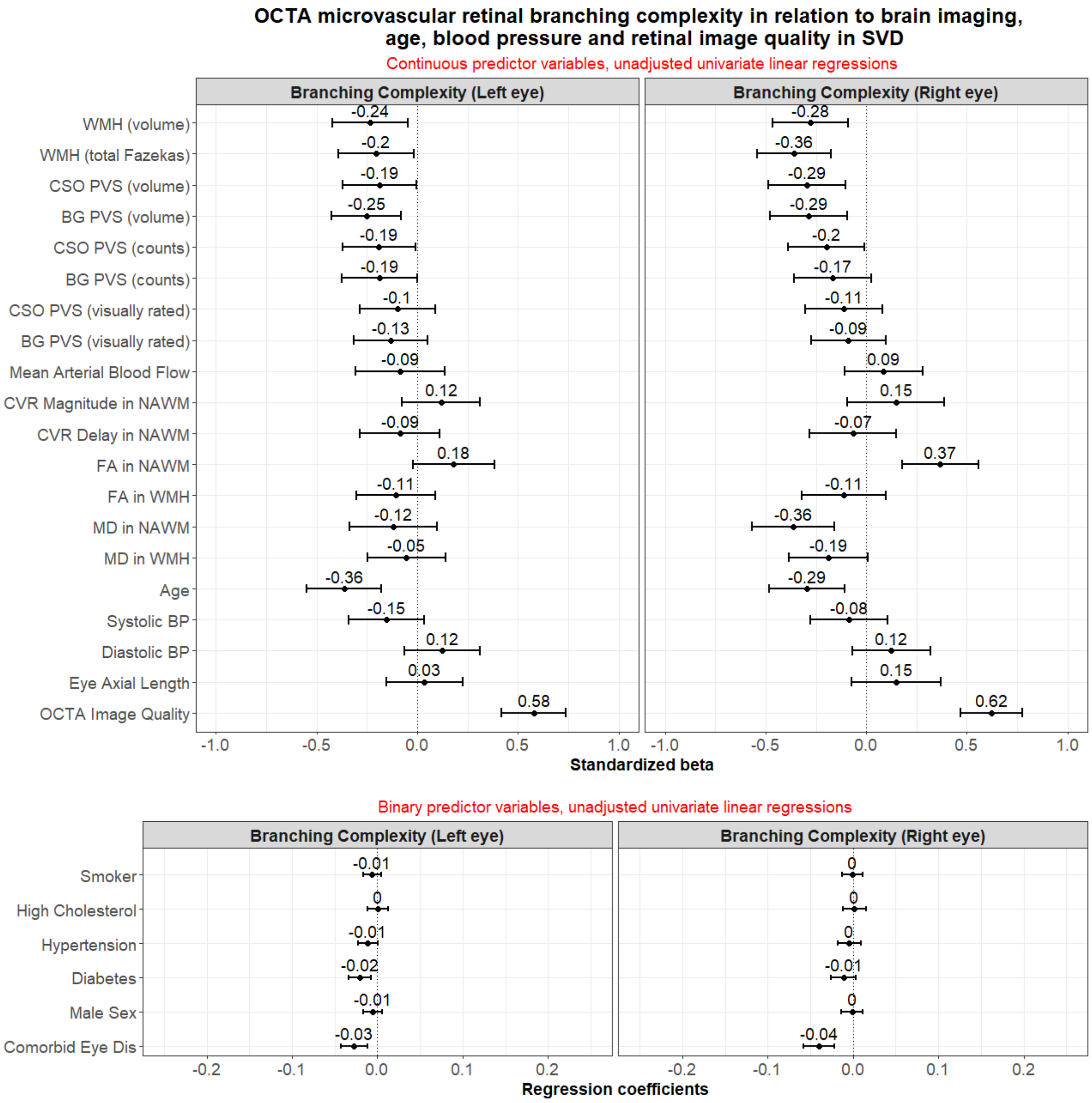

Supplementary Figure S3. Unadjusted univariate models. OCTA vessel branching complexity in relationship to age, sex, vascular risks and brain imaging measures of SVD showing standardized betas for continuous predictors and regression coefficients for binary predictors. *BG PVS = basal ganglia perivascular spaces; CSO PVS = centrum semiovale perivascular spaces; CVR = cerebrovascular reactivity; FA = fractional anisotropy; MD = mean diffusivity; OCTA = optical coherence tomography angiography; SVD = small vessel disease; WMH = white matter hyperintensities.*

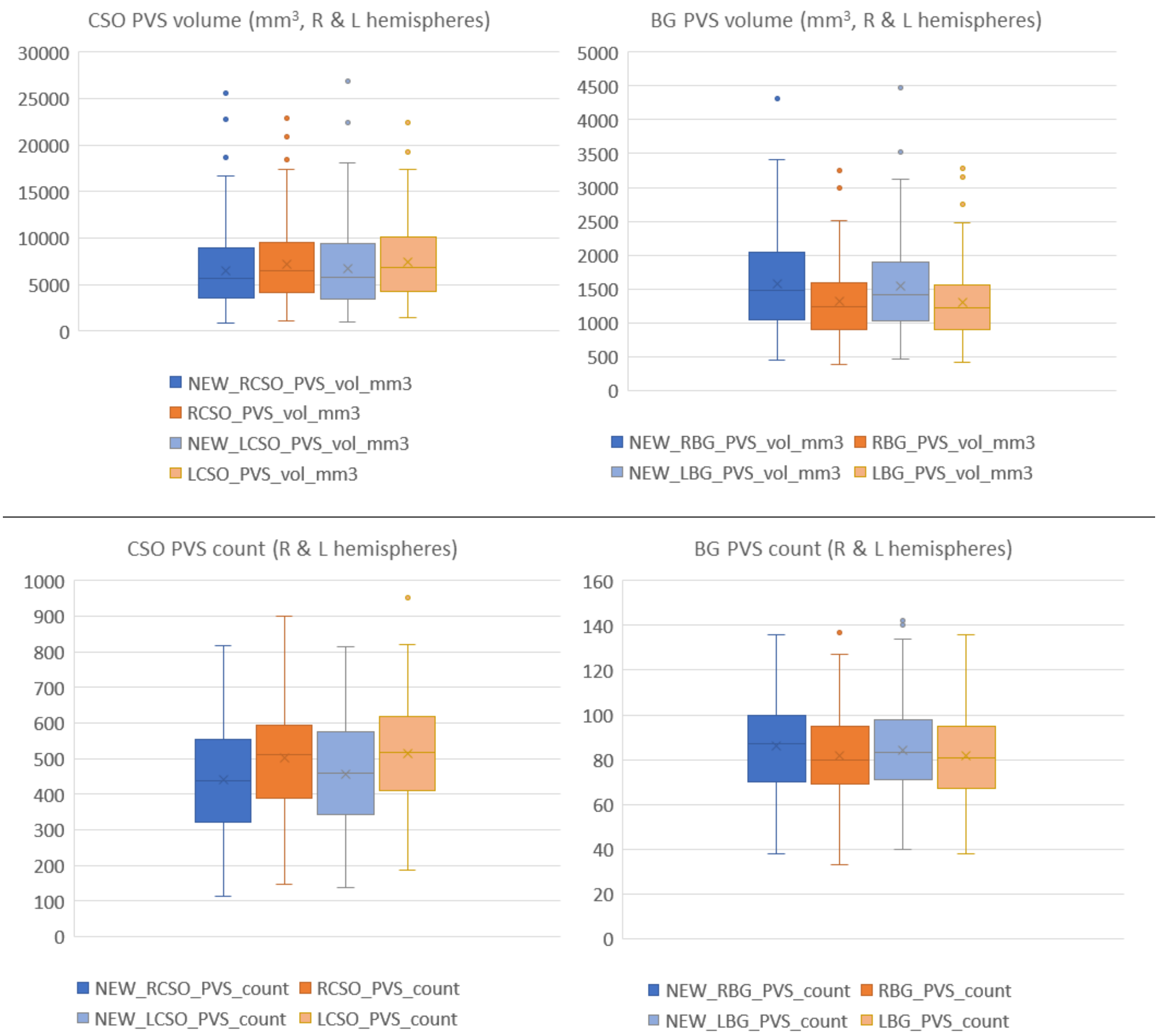

Supplementary Figure S4. PVS counts and volume resultant from using a first set of thresholds (orange box plots) and a second set (blue box plots, labelled as "NEW").

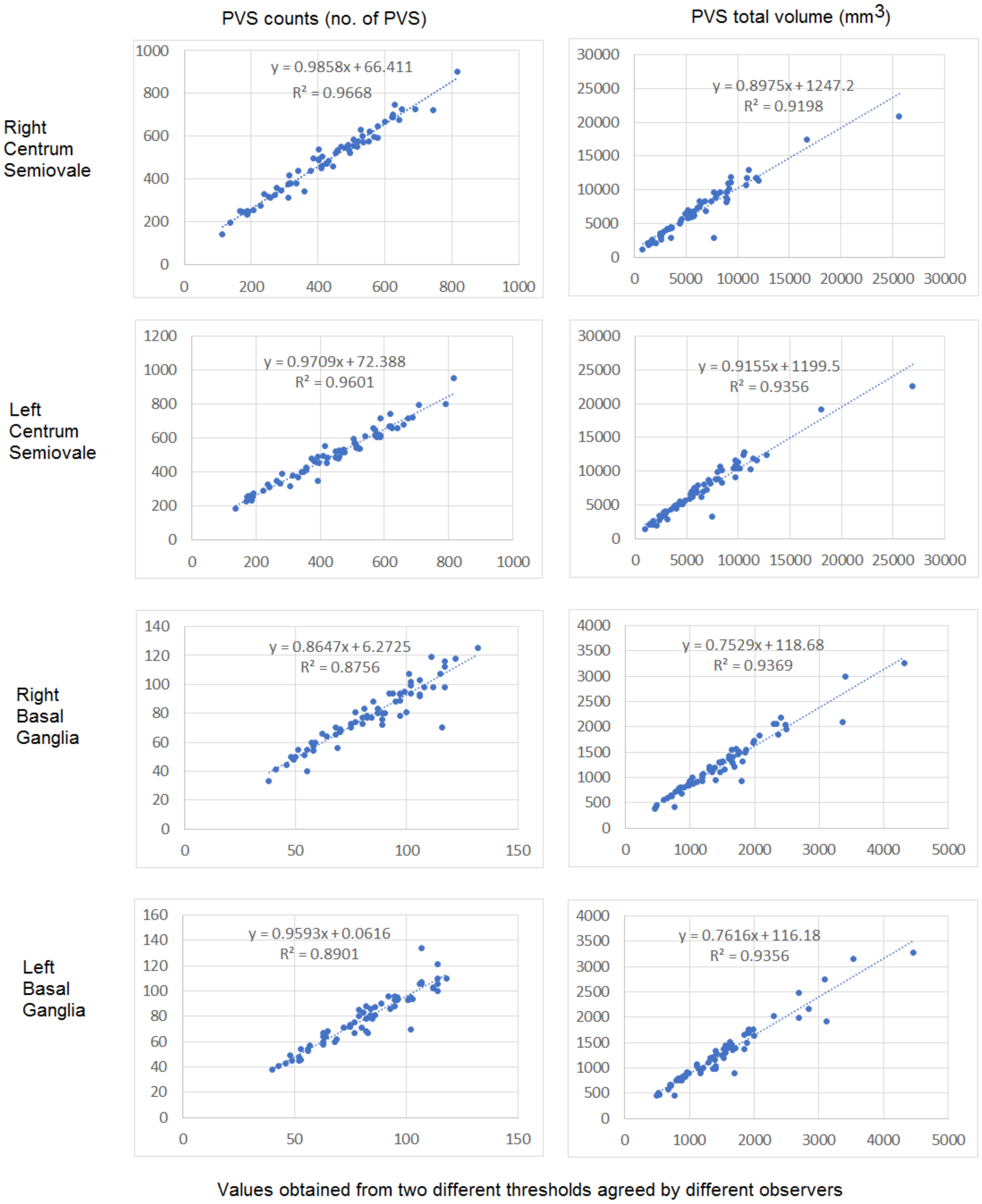

Supplementary Figure S5. Relationship between the raw PVS volumes and counts obtained by both sets of thresholds.

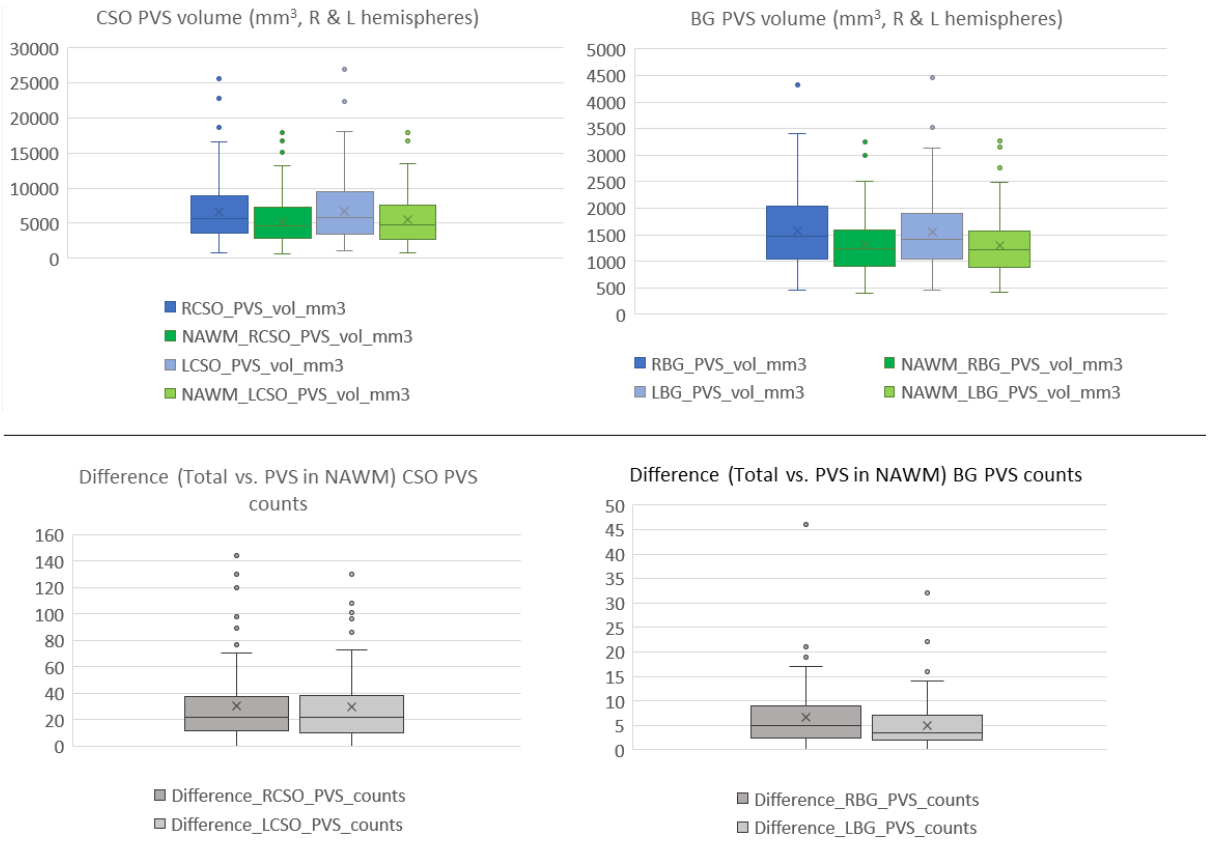

Supplementary Figure S6. Difference between PVS counts and volumes assessed in the entire ROIs using the second set of thresholds, and the PVS counts and volumes assessed only in the normal tissues (NAWM stands for normal appearing white matter).

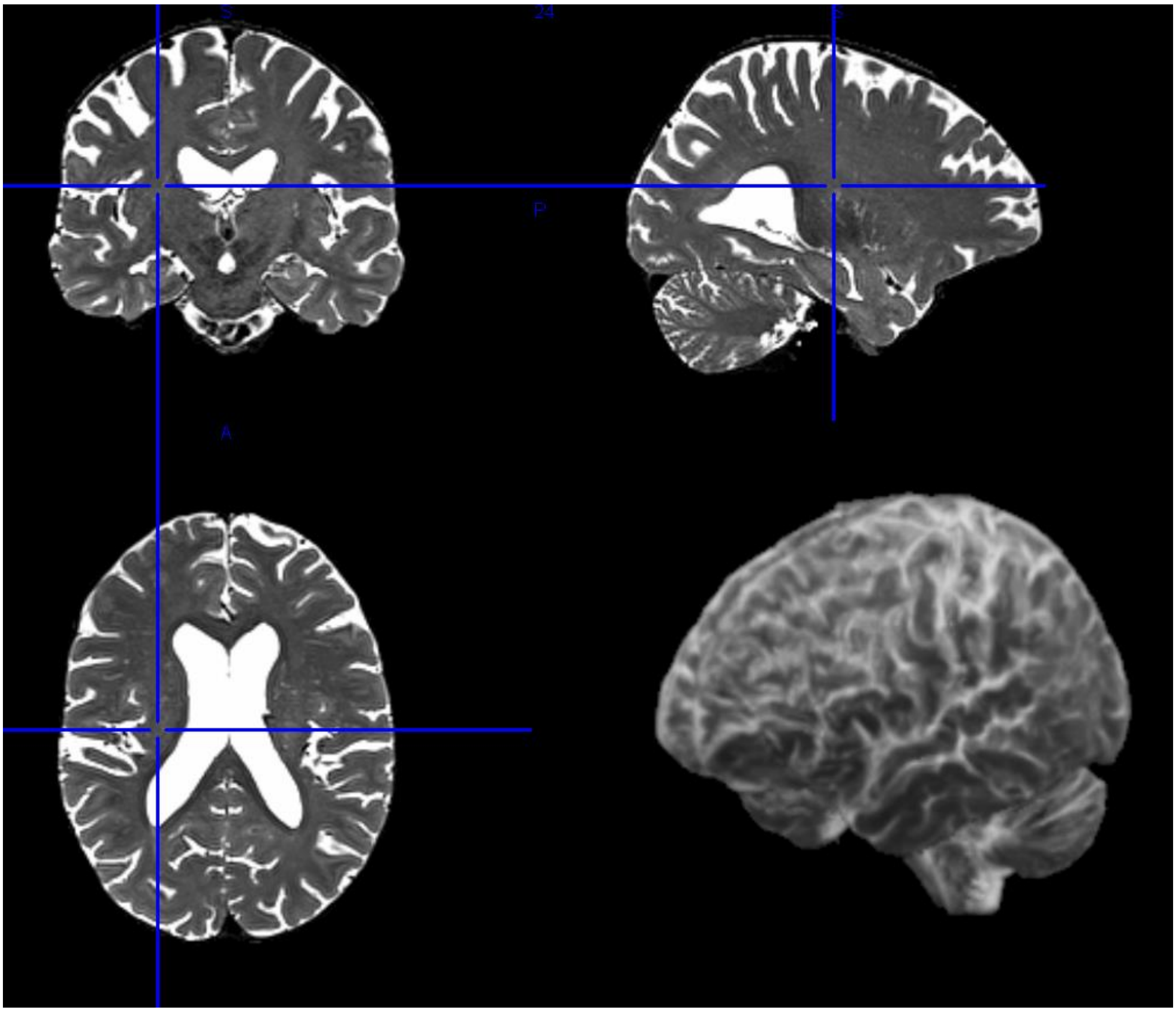

Supplementary Figure S7a. Example of PVS segmentation – low burden (without PVS masks).

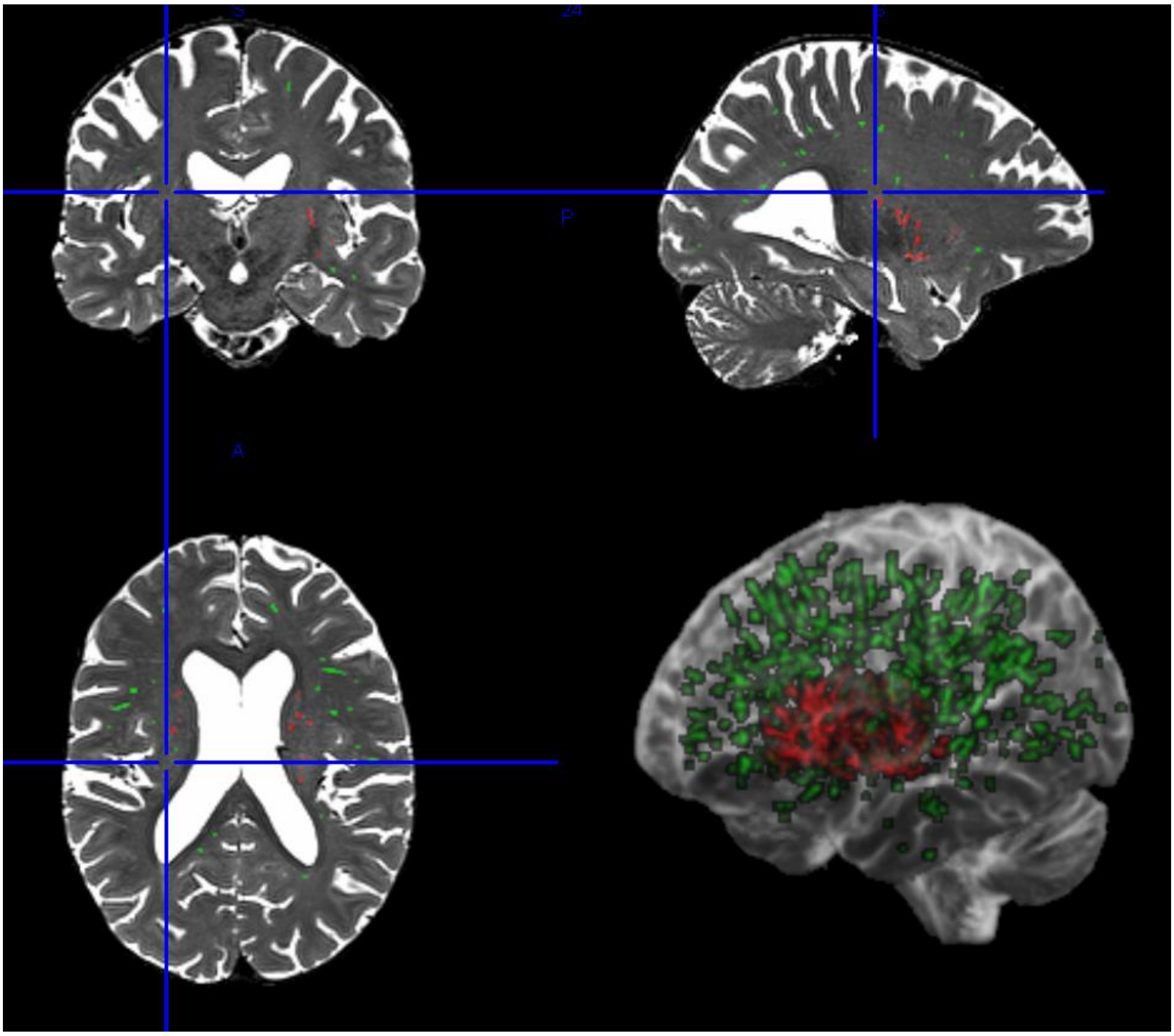

Supplementary Figure S7b. Example of PVS segmentation – low burden (with PVS masks).

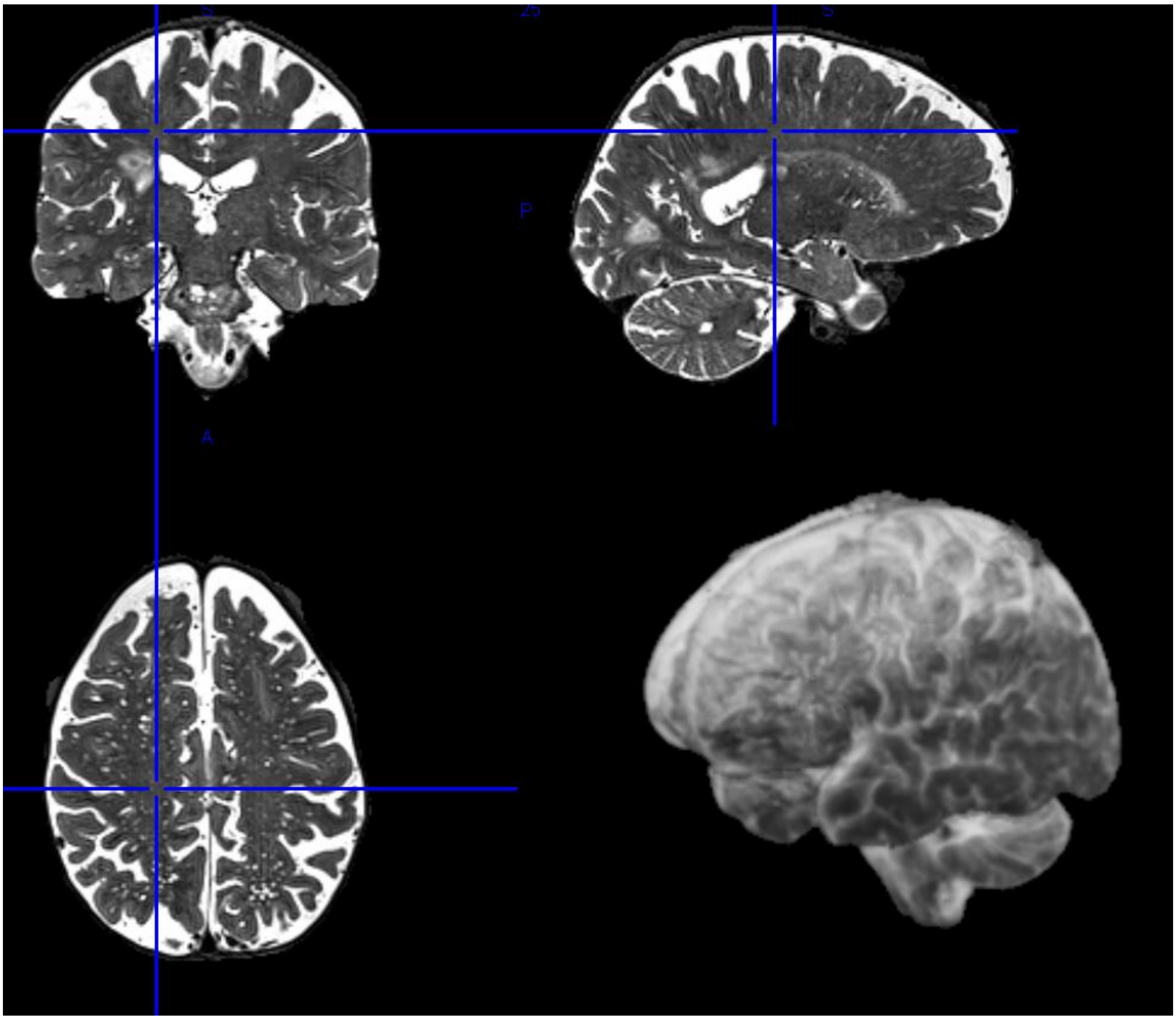

Supplementary Figure S8a. Example of PVS segmentation – high burden (without PVS masks).

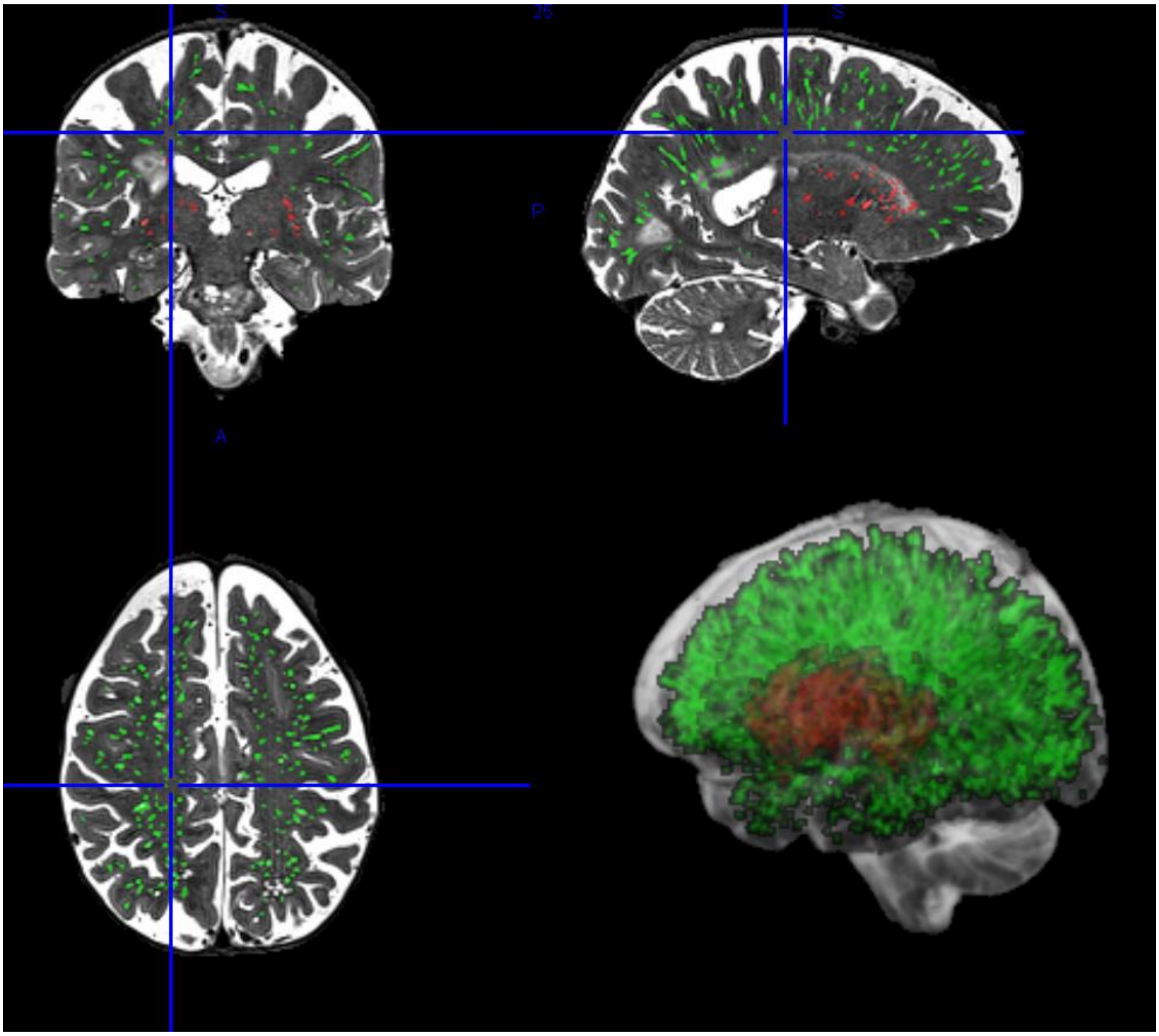

Supplementary Figure S8b. Example of PVS segmentation – high burden (with PVS masks).

References

1. Wardlaw JM, Smith EE, Biessels GJ, et al. Neuroimaging standards for research into small vessel disease and its contribution to ageing and neurodegeneration. Lancet Neurol. 2013;12:822–38. doi:10.1016/S1474-4422(13)70124-8.

2. Fazekas F, Chawluk JB, Alavi A, et al. MR signal abnormalities at 1.5 T in Alzheimer' s dementia and normal aging. Am J Roentgenol. 1987;149(August):351–356. http://www.ajronline.org/cgi/content/abstract/149/2/351.

3. Potter GM, Chappell FM, Morris Z, et al. Cerebral perivascular spaces visible on magnetic resonance imaging: development of a qualitative rating scale and its observer reliability. Cerebrovasc Dis. 2015;39:224–31.

4. Potter G, Morris Z, Wardlaw J. Enlarged perivascular spaces (EPVS): a visual rating scale and user guide. Available at: https://www.ed.ac.uk/files/imports/fileManager/epvs-rating-scale-user-guide.pdf.

5. Wardlaw JM, Benveniste H, Nedergaard M, et al. Perivascular spaces in the brain: anatomy, physiology and pathology. Nat Rev Neurol 2020; 16(3): 137–153. doi:10.1038/s41582-020-0312-z

6. Jenkinson M, Bannister P, Brady M, et al. Improved optimisation for the robust and accurate linear registration and motion correction of brain images. NeuroImage. 2002;17,825– 841.

7. Zhang Y, Brady M, Smith S. Segmentation of brain MR images through a hidden Markov random field model and the expectation-maximization algorithm. IEEE Trans Med Imaging. 2001;20:45–57. doi:10.1109/42.906424.

8. Pechaud M, Jenkinson M, Smith S. BET2 - MRI-based estimation of brain, skull and scalp surfaces. FMRIB Tech Rep TR06MP1. 206AD. https://www.fmrib.ox.ac.uk/datasets/techrep/tr06mp1/tr06mp1.pdf.

9. Valdes Hernández MDC, Ferguson KJ, Chappell FM, et al. New multispectral MRI data fusion technique for white matter lesion segmentation: method and comparison with thresholding in FLAIR images. Eur Radiol. 2010;20:1684–1691. doi:10.1007/s00330-010-1718-6

10. Wen W, Sachdev P. The topography of white matter hyperintensities on brain MRI in healthy 60- to 64-year-old individuals. NeuroImage 2004; 22(1): 144-154. doi:10.1016/j.neuroimage.2003.12.027

11. Ballerini L, Lovreglio R, Valdés Hernández M del C, et al. Perivascular spaces segmentation in brain MRI using optimal 3D filtering. Sci Rep. 2018;8:1–11. doi:10.1038/s41598-018-19781-5

12. Ballerini L, Booth T, Valdés Hernández M del C, et al. Computational quantification of brain perivascular space morphologies: Associations with vascular risk factors and white matter hyperintensities. A study in the Lothian Birth Cohort 1936. NeuroImage Clin. 2020;25:102120. doi:10.1016/j.nicl.2019.102120

13. Barnes A, Ballerini L, Valdes Hernandez MDC, et al. Topological relationships between perivascular spaces and progression of white matter hyperintensities: A pilot study in a sample of the Lothian Birth Cohort 1936. Front Neurol. 2022;13:889884. doi: 10.3389/fneur.2022.889884

14. Clayden JD, Munoz Maniega S, Storkey AJ, et al. TractoR: Magnetic resonance imaging and tractography with R. J Stat Softw. 2011;44(8).

15. Smith SM. Fast robust automated brain extraction. Human Brain Mapp. 2002;155:143–155. doi:10.1002/hbm.10062

16. Andersson JLR, Skare S, Ashburner J. How to correct susceptibility distortions in spin-echo echo-planar images: application to diffusion tensor imaging. NeuroImage. 2003;20:870–888. doi:10.1016/S1053-8119(03)00336-7

17. Andersson JLR, Sotiropoulos SN. An integrated approach to correction for off-resonance effects and subject movement in diffusion MR imaging. NeuroImage. 2016;125:1063–1078. doi:10.1016/j.neuroimage.2015.10.019

18. Pierpaoli C, Basser PJ. Toward a quantitative assessment of diffusion anisotropy. Magn Reson Med. 1996;36(6):893–906. doi:10.1002/mrm.1910360612

19. Bernal J, Valdes Hernandez MDC, Escudero J., et al. Assessment of perivascular space filtering methods using a three-dimensional computational model. Magn Reson Imaging. 2022;93:33–51. https://doi.org/10.1016/j.mri.2022.07.016
